# The European strategy for accelerator-based photon science

**DOI:** 10.1140/epjp/s13360-023-03947-w

**Published:** 2023-04-25

**Authors:** R. Abela, C. Biscari, J. Daillant, H. Dosch, L. Rivkin

**Affiliations:** 1 Paul Scherrer Institute, Forschungstr. 111, 5232 Villigen, Switzerland; 2 ALBA Synchrotron, Carrer de la Llum 2-26, 08290 Cerdanyola del Vallés, Spain; 3 Synchrotron SOLEIL, L’Orme des Merisiers, 91190 Saint-Aubin, France; 4 Deutsches Elektronen-Synchrotron DESY, Notkestr. 85, 22607 Hamburg, Germany

## Abstract

The League of European Accelerator-based Photon Sources (LEAPS), comprising 19 large-scale user facilities in 10 member and associated states, has put forward for the first time a European strategy for a transformative way of cooperation, thereby mobilizing the members’ substantial expertise in photon science and technology, in infrastructure management and service to users and stakeholders. This European Strategy for Accelerator-based Photon Sources—ESAPS 2022—is a coherent pan-European plan addressing the future challenges and needs of the new era in research and innovation, designed to put Europe in a global leadership position in important future key technologies. In ESAPS2022, ambitious facility upgrades and technology development plans as well as a new strategic challenge-driven use of these facilities are discussed.

## Introduction

Accelerator-based synchrotron radiation facilities, i.e. high-brightness storage ring sources and free electron lasers (FELs), provide unique analytic tools for decoding matter. They cover a wide range of areas such as the destruction-free *operando* and in situ exploration of the molecular and electronic structure as well as processes in novel materials, static and dynamic structural studies of biological systems as well as high resolution imaging of complex systems in a holistic way. Today, 19 facilities sited throughout Europe are providing access to more than 35.000 researchers from academy and industry across all scientific disciplines from materials characterization, drug design, biochemistry, health, catalysis, geosciences and planetary research to palaeontology and cultural heritage.

### LEAPS foundation, ambition, strategic relevance

The European Accelerator-based Photon Facilities have joined forces in 2017 through the formation of the strategic European consortium LEAPS [1] (League of European Accelerator-based Photon Sources). LEAPS´ goal is to work collaboratively in a new strategic way, mobilizing the members’ substantial expertise in photon science and technology, in infrastructure management and service to users and stakeholders. One of the consortium’s ambitions is to become a driving force in the formation of European teams with state-of-the-art scientific and technological competences that can leverage the full potential of the LEAPS facilities meeting the urgent needs and societal challenges of the next decades.

LEAPS facilities are based on the most modern technologies available today and thus constitute a key component of the European high-tech strategy. They cover critical core competences for an advanced society in a dynamic international competition. During the years of the COVID-19 pandemic, the relevance of this new research consortium has become particularly visible, as all LEAPS facilities have made their experimental stations available to virologists, drug designers, and hospitals for precision structural analyses. Among the future challenges we will have to solve in Europe, are combating vexing diseases and climate change and designing a sustainable circular economy.

### Infrastructure and technology developments

During recent years, the LEAPS consortium, and its user community, which includes the LEAPS Strategic Partner ESUO [2] (European Synchrotron and FEL Users Organization), the national users associations, and the facility users’ associations, have defined measures for improving the technical capabilities of the facilities to comply with the future need of European researchers from academia and industry.

These include the following aspects:

- The upgrade of the storage rings with revolutionary novel magnetic lattice based on the so-called multi-bend achromat technologies which delivers an unprecedented new level of brilliant radiation and the further development of the novel FEL facilities with emerging concepts (continuous wave operation, external laser-and self-seeding, new detection and computing capabilities)
- Continuous development of FELs, aiming at better control of spectral and temporal properties to match the requirements for extreme time resolution down to attosecond level, high repetition rates, better time synchronization with external lasers, increased peak intensity and improved longitudinal coherence.
- The development of enabling technologies encompassing advanced instrumentation for beam control and diagnostics; novel optics to preserve the beam quality during beam transport to the experiment; novel high-throughput sample delivery systems; advanced pixel detectors for highest data rate and in particular novel AI-assisted data handling and real-time data evaluation and simulation technologies

The costs for the facility upgrades are substantial and are covered by the national funding agencies of the LEAPS facilities. One aim of these upgrade plans is—against the background of climate change—to dramatically reduce facilities’ electrical power consumption and reduce their carbon footprint.

### Service provision for European researchers from academia and industry

LEAPS facilities offer open access to national and international researchers inviting discovery driven as well as applied research projects with an emphasis on inviting young investigators at the beginning of their career to exploit these high-tech infrastructures for their research. Access to the facilities is granted after a successful review process, where the incoming proposals are rigorously ranked by excellence and relevance.

LEAPS facilities provide over 1 million hours per year of user access to researchers from academia and industry. The funding for the access to the LEAPS facilities is provided primarily by national funding agencies and has in the past been also supported by the European Commission (“transnational access, TNA”). The facilities are also available to industrial partners free of charge when the results are made public. In the case of proprietary research projects, beamtime hours are charged to the industrial users at full cost.

### European strategy for accelerator-based photon sources

The requirements for photon-based analytical tools to make significant contributions to science and innovation imply the implementation of extensive technology developments. These in turn require the formulation of a new European strategy for the further development of these research infrastructures and their future use.

LEAPS has formulated a European Strategy for Accelerator-based Photon Sources—ESAPS 2022—as a coherent plan addressing the future challenges and needs of the new era in research and innovation, designed—against the background of the current global challenges—to put Europe in a global leadership position in this important technology of the future. This strategic document has been presented to representatives of the European Commission and Parliament on May 31, 2022.

It encompasses:

- The upgrade of synchrotron radiation facilities implementing a disruptive high-brilliance electron lattice
- The further development of the emerging FEL technologies
- Joining forces for enhancing facility operation by implementing new digital technologies including digital twin concepts and artificial intelligence tools.
- The expansion of service provision to speed-up emerging research for societal challenges and enabling new strategic long-term cooperation with European Partnerships (also increasing resilience).

## Future service provision

### Future schemes of service provision

As a response to the grand challenges of today and to the future needs of European researchers from academia and industry, the LEAPS consortium is expanding its service provision in the following ways:

- *The facilities will maintain* the established discovery-driven service provision supporting and developing new, disruptive ideas:

The development of a new generation of dramatically more brilliant sources enables high-throughput spectroscopic and structural characterization to be fully integrated using Artificial Intelligence methods into the materials synthesis workflow, together with simulations. It has the potential of a tremendous acceleration in the discovery process, with the leverage of the transformative upgrades, instrument developments and the establishment of powerful partnerships with the European researchers. Importantly, any investment at the facility level benefits all the above challenges making it a most cost-effective way to support discovery-driven research.

(b) *The facilities will implement* a novel targeted challenge-driven service provision:

In the first amendment of the LEAPS declaration of 2017 [3], its facilities are opened to strategic access by large European partnerships and initiatives which address Horizon Europe missions [4] and require tailor-made access to LEAPS facilities for carrying out their long-term ambitious research projects. The large projects and research consortia stand to benefit from the well-established peer-review process at the LEAPS facilities which guarantees highest standard of scientific excellence. As already tested by some facilities, going further can be achieved by providing dedicated beamtime, introducing specific criteria in the proposal selection process.

(c) *The facilities will develop* a new remote service provision:

An integral part of LEAPS’ European strategy includes the enhanced automated operation of experimental schemes, remote access for users, including on-line data analysis in near real time to guide an ongoing experiment. The resulting reduction in unnecessary travel will contribute to the reduction of the carbon footprint of the future user operation.

### Future pilots of strong partnerships addressed by targeted challenge-driven service provision

In the coming years, LEAPS sees its role particularly in strategic collaboration with leading European players aiming at accelerating research, notably in the areas of:

- Public health challenges
- Rational catalyst design
- Green hydrogen
- Water-based technologies
- Quantum technologies and advanced manufacturing

## Facility upgrades and key technologies

### Facility upgrades

By means of a transformative pan-European cooperation, LEAPS members are embarking on a journey to enhance European competitiveness in the development of key technologies in a fiercely competitive global world.

There has never been a more important time to do this, both because of the urgency of the societal challenges themselves, and because of the massive investment that is boosting the performance and delivery of such facilities elsewhere in the world.

During the past decades, many European countries have built accelerator-based photon sources using the most advanced technologies known at that time [5]. Due to the experience gained during the operation of the storage rings and the improved technological capabilities of the facilities and industry, a revolutionary upgrade of the storage ring layout as well as the photon beamlines has been initiated at MAX IV (Sweden, Lund) with the multiple-bend-achromat (MBA) technology followed by the ESRF (France, Grenoble) with a new hybrid multiple-bend-achromat (HMBA) technology reaching an unprecedented level of photon beam brightness and degree of coherence, allowing new types of experiments. These achievements have prepared the ground for a further upgrade programme of many of the national-based storage rings in Europe and have positioned Europe very clearly at the forefront of this technology.

The FEL facilities, pioneered in Europe, are internationally at the forefront of technology and science. Nevertheless, new projects and upgrades are envisaged for the next decade enhancing their capabilities as well as introducing new accelerating schemes providing ultrashort, phase-locked X-ray beams.

The European Strategy will be implemented as an open innovation effort in strong collaboration with industry stakeholders who are aiming to extend their product portfolios and markets. Their participation will certainly boost innovative industrial research and improve their competitiveness in the global market.

The implementation of key enabling technologies will envisage new avenues for smart specialization, integrating all stakeholders across Europe and thereby also contributing to the European cohesion process. This includes complementary solutions and specialized European consortia in high-tech developments.

For this purpose, LEAPS has launched a comprehensive analysis of the specific expertise in key technologies currently developed in the LEAPS facilities with a critical mass. It encompasses the development of new schemes in X-ray detectors, X-ray optics, dedicated sample environments, photon source development and advanced information technologies.

The timeline for the upgrades is an essential issue. Obviously, this will depend critically upon the funding decisions. These activities must be coordinated to secure that the other operating facilities can partially accommodate the users from a facility that is upgrading. These coordinated actions make part of the roadmap efforts.

**Fig. 1:**
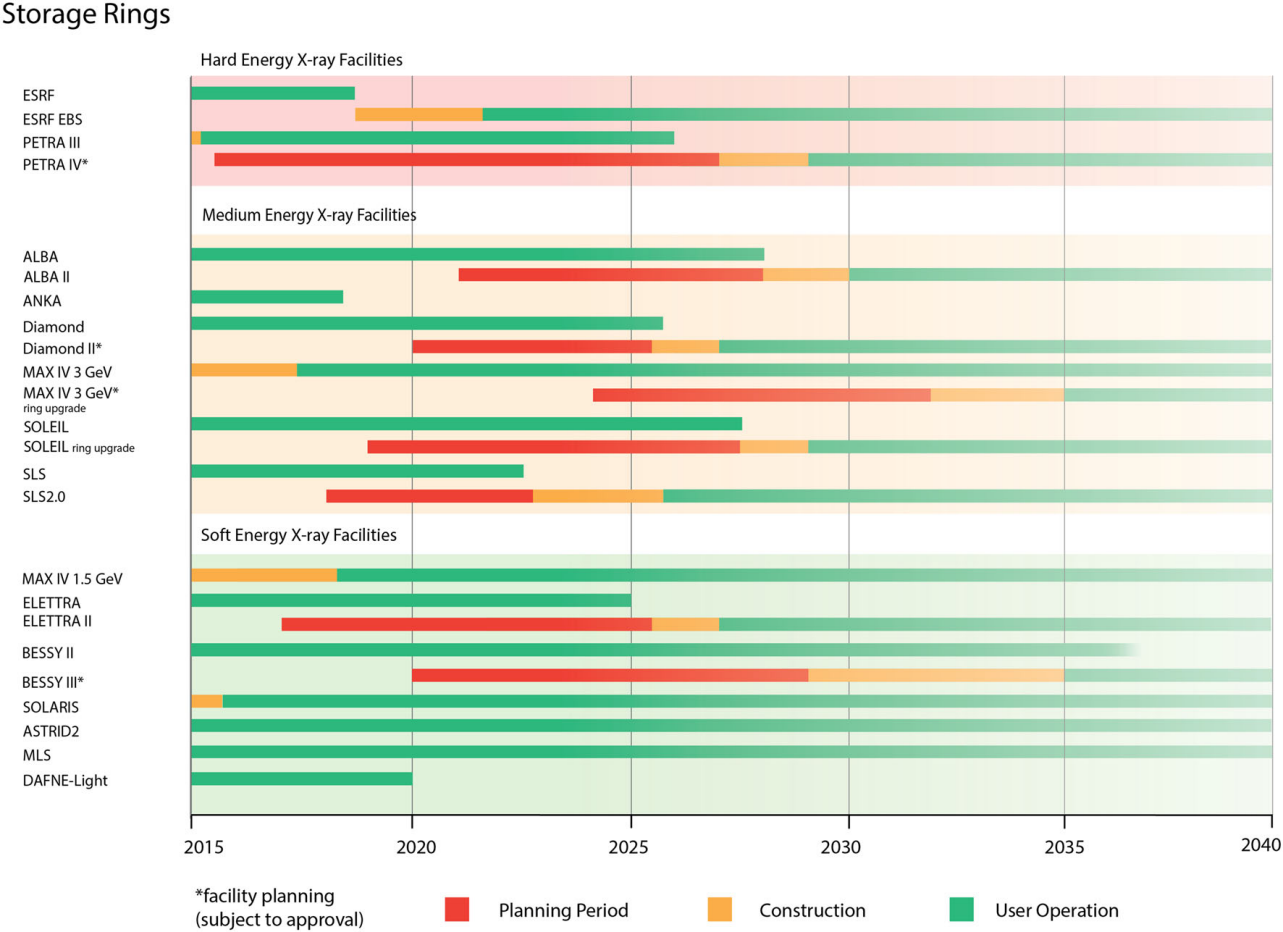
Timeline of the existing storage ring facilities, approved upgrades and plans for upgrades not yet approved (marked with an asterisk)

**Fig. 2:**
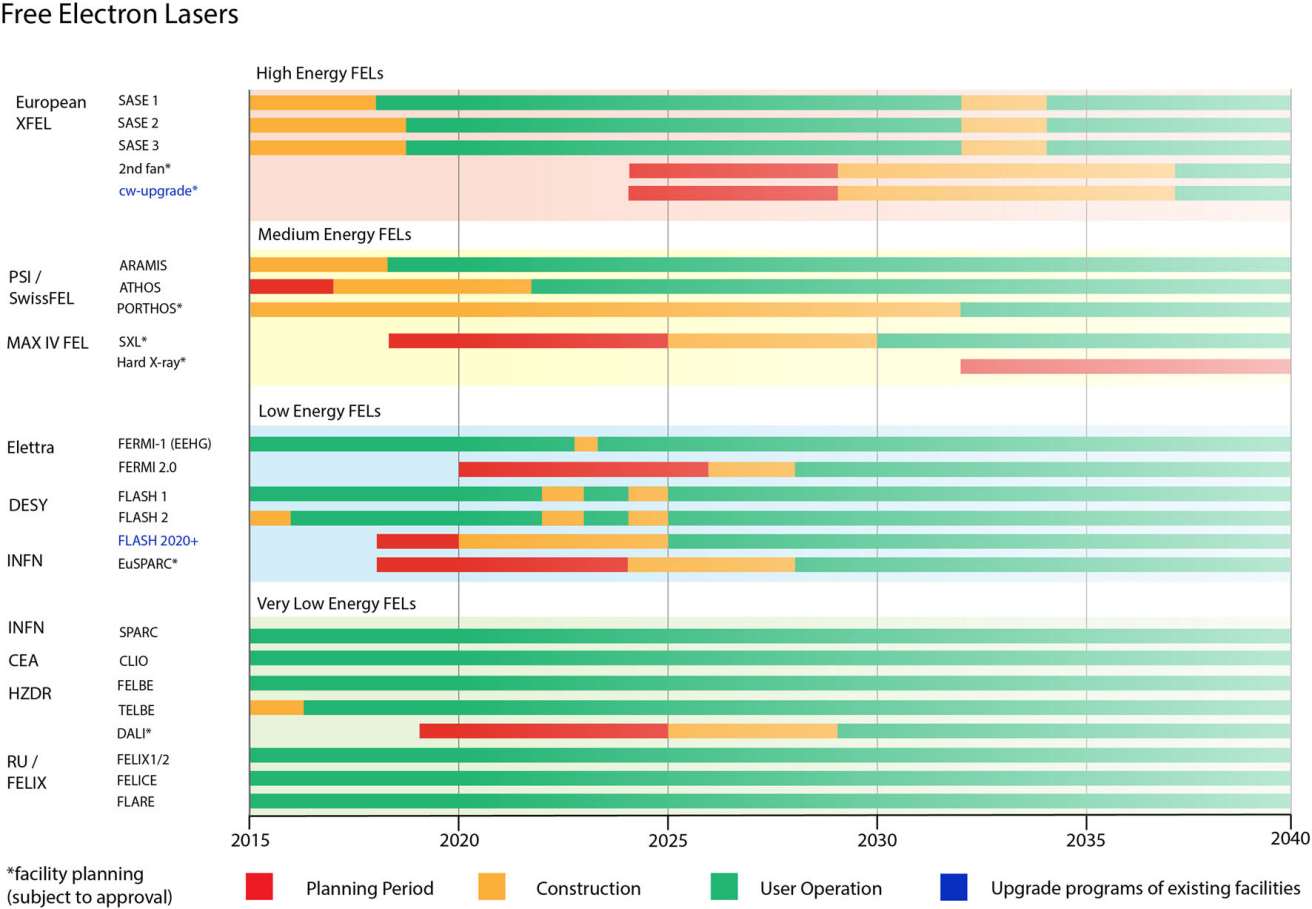
Timeline of the existing FEL facilities, approved upgrades and plans for upgrades not yet approved (marked with an asterix)

Facility plans, including the upgrades, depicted in Figs. 1 and 2, are being developed by the corresponding owner institutions, keeping as a reference to the global framework. The detailed description of such plans, the scientific goals and complementing information are the focus of this topical issue.

As it has been anticipated above, the next major improvement for storage ring light facilities is the rebuilding of the accelerator lattice to reduce the electron beam emittance substantially. This results in diffraction limited photon beams at least for photon energies up to the keV regime.

**Fig. 3:**
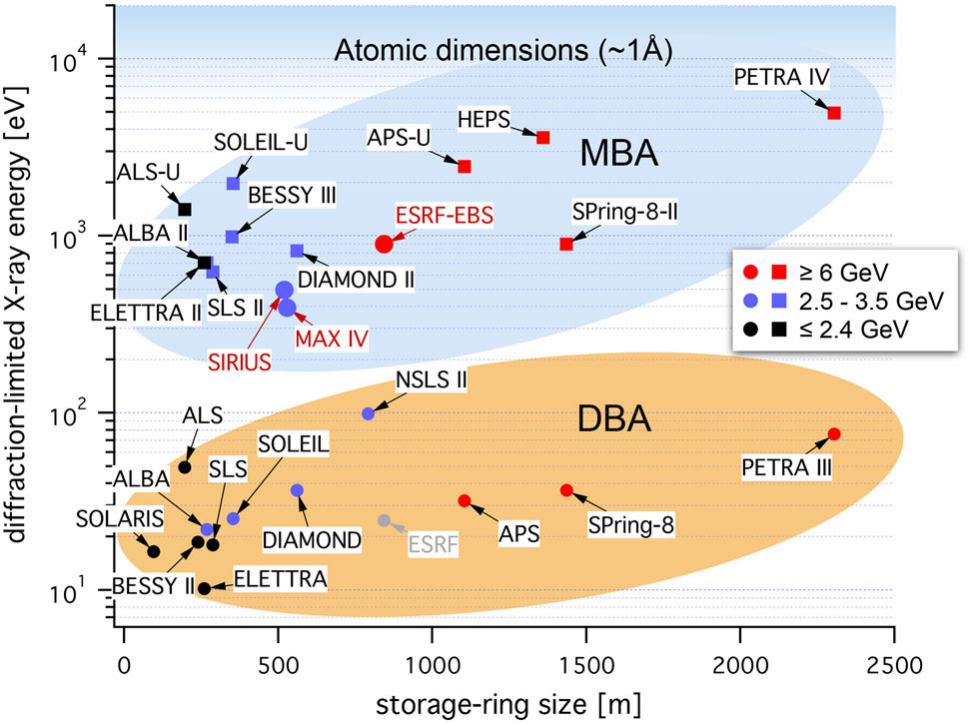
Diffraction limited photon energy (defined as hc/4πeεx) reached at various existing facilities (full circles) and after planned upgrades (full squares), including some selected extra-European ones. The orange and blue islands denote the disruptive change in accelerator technology in going from the double/triple bend achromat lattice to the newer multi-bend achromat storage ring design. Data are supplied by the different facilities, adapted by C.G. Schroer

The following Fig. 3 shows the photon energy where the diffraction limit is reached of present storage ring facilities around the world and the potential improvements when the upgrades are implemented.

## Digital transformation of the operation of European user facilities

### Development of advanced digital technologies

For the future, LEAPS facilities need a sustainable operation, the development of tools to be used by non-expert users groups and, last but not least, to become more resilient in times of massive constraints on operations and mobility. This requires a fundamental reconsideration of user operation including remote operation tools and artificial intelligence concepts in the entire operation of the experimental installations from the accelerator to big data handling.

The DIGITAL LEAPS initiative pursues the development of a digital interface system to access and autonomously operate green facilities, via digital twins, artificial intelligence and machine learning, virtual diagnostics, androids for remote access, and the design of further photon instruments for remote access and standards for fully automated user beamlines.

The above-mentioned initiatives are tightly connected with a pathway to more green operation of the LEAPS facilities. Digital remote user operation is going in the direction of rationalizing the movements of the people and negative impact on the environment. At the same time, it will open up new access possibilities from geographically far regions, i.e. Africa or Latin America. Smart user network and digital collaboration platforms will multiply the possibilities with common developments in the areas of environmental and neutral climate challenges.

### Towards open science

Large-scale research infrastructures produce massive amounts of scientific data daily. Today, the amount of data doubles every year, and the increase in volume is expected to continue. This poses storage and (re)usability challenges, which are best addressed by the ‘FAIR’ paradigm: Findable, Accessible, Interoperable, and Re-usable.

Recognising the importance of fostering open science and open innovation, the EC supported the idea of a European Open Science Cloud (EOSC) that took shape in 2015.

Two projects, PaNOSC [6]and ExPaNDS [7], were funded by the European Commission to develop the EOSC for the photon and neutron community. The outcomes of these two projects have been the publication of open data, the development of federated open data catalogues, EOSC-ready community Authentication and Authorization Infrastructure (AAI), and services for remote data analysis, simulation, and an e-learning platform. These outcomes are necessary for the adoption of FAIR data practices and to fully exploit data coming out of the LEAPS facilities.

LEAPS has put large efforts into realizing the idea of open science beyond the EOSC clusters PaNOSC/ExPaNDS by supporting the FAIR principles of data produced at their facilities also by nationally funded initiatives. To capitalize on these efforts towards open science, investment in sustaining and strengthening open science activities is further needed to develop a photon and neutron PaN Open Data Commons as an essential component of EOSC for showcasing and accessing data.

The PaN Open Data Commons will enable new user communities to access and exploit the unique data being produced at the LEAPS facilities to do new science, e.g. the Human Organ Atlas [8] is revolutionizing digital histology and medical research with high resolution 3D volumes of complete human organs.

## Transversal priority action items

Today, the European users of synchrotrons and FELs are an open and extremely innovative community, growing strongly, in part due to the increasing number of users from Eastern European countries. LEAPS facilities are meeting hubs for international, interdisciplinary, and intermixed scientific groups and highly interlinked with neighbouring research infrastructures.

### Training

LEAPS members are a perfect mix of research infrastructures and user facilities. Highly motivated and experienced staff develop and operate instruments, pushing the technology limits beyond state of the art to conduct experiments at the forefront of science, blending technical knowledge with an awareness of the scientific challenges nurtured by the extensive user community, with whom the interaction is a continuous enrichment process in both directions. The multiple ERC grants addressing the evolution of methods at LEAPS RIs or accessing them for their unique analytical capabilities are successful examples of the fertile environment for scientific job developments.

Specific education and training, as well as dedicated career development programmes, will sustain and develop such staff. LEAPS proposes a multidisciplinary European curriculum targeting scientists, technicians and engineers as well as future managers. It will include mobility and exchange programmes and mentoring of the early career staff. It will be balanced in terms of diversity.

Fostering innovation needs strengthening bridges between the academic and the industrial world. Development of careers sharing years of staging in both environments will help in creating a common language, and in providing, for public researchers and engineers a business vision of applied research, and an innovation vision to industrial staff.

### European synchrotron and free electron laser user organisation

The European Synchrotron and Free Electron Laser User Organisation (ESUO), founded in 2010, represents the interests of 25,000 + users of SR and FEL facilities in Europe through delegates from 30 nations nominated by national/facility user organisations or equivalent and via an 8-member Executive Board. ESUO’s vision is to support a thriving European synchrotron and FEL user community with equal opportunities of access and participation for all scientists based solely on the scientific merit of their ideas. ESUO’s mission envisages achieving continued unencumbered access to SR and FEL research infrastructures, ideally eliminating geographic or financial barriers in user participation, and with as simple an access model as practicable. In addition, ESUO seeks to improve gender balance among users, foster contacts and share knowledge with users from widening countries, co-develop actions for advanced techniques and beamlines with LEAPS, and also to collaborate with user organisations of other analytical facilities. ESUO has been a LEAPS Strategic Partner since 2021.

### Synergies with neighbouring research infrastructures

LEAPS facilities offer a rich diversity of analytical tools based on the interaction of synchrotron and FEL radiation with matter. The complexity of understanding properties of matter is also addressed by other research infrastructures, which make use of different and complementary probing instruments and methods.

In 2020, LEAPS initiated the creation of the network of Analytical Research Infrastructures in Europe (ARIE) [9]—all together providing unique windows into the workings of the world around us. ARIE includes the powerful photon sources, such as storage rings, FELs and laser systems; sources of protons for beam-therapy, of neutrons and of ions; and facilities dedicated to advanced electron-microscopy and high-magnetic fields, altogether comprising more than one hundred facilities. This network of research infrastructures, unique in the world, enables LEAPS to react quickly and effectively to address societal challenges as a strong stakeholder within the European Research Area.

## Concluding remarks

The European Strategy ESAPS 2022 charts a route into a future that features environmentally friendly technologies and research strategies that support solving societal challenges while making a critical contribution keeping Europe at the international forefront of research and development.

The European Strategy ESAPS 2022

- *Supports* high quality scientific research in Europe.
- *Contributes* the development of the skills of the next generation of scientists and engineers.
- *Devises* the particle accelerators and associated technologies of tomorrow for a wide range of use in manufacturing and service industries in health, materials design, energy and security.
- *Supports* European industry in new product and market development and by accelerating product design and development.

## Data Availability

No Data associated in the manuscript.
